# Evolution and origin of merlin, the product of the *Neurofibromatosis type 2 *(*NF2*) tumor-suppressor gene

**DOI:** 10.1186/1471-2148-5-69

**Published:** 2005-12-02

**Authors:** Kseniya Golovnina, Alexander Blinov, Elena M Akhmametyeva, Leonid V Omelyanchuk, Long-Sheng Chang

**Affiliations:** 1 Institute of Cytology and Genetics, Russian Academy of Sciences, 10 Lavrent'ev Ave., 630090, Novosibirsk, Russia; 2 Center for Childhood Cancer, Children's Research Institute, Children's Hospital and Department of Pediatrics, The Ohio State University, 700 Children's Drive, Columbus, OH 43205-2696, USA

## Abstract

**Background:**

Merlin, the product of the *Neurofibromatosis type 2 *(*NF2*) tumor suppressor gene, belongs to the ezrin-radixin-moesin (ERM) subgroup of the protein 4.1 superfamily, which links cell surface glycoproteins to the actin cytoskeleton. While merlin's functional activity has been examined in mammalian and *Drosophila *models, little is understood about its evolution, diversity, and overall distribution among different taxa.

**Results:**

By combining bioinformatic and phylogenetic approaches, we demonstrate that merlin homologs are present across a wide range of metazoan lineages. While the phylogenetic tree shows a monophyletic origin of the ERM family, the origin of the merlin proteins is robustly separated from that of the ERM proteins. The derivation of merlin is thought to be in early metazoa. We have also observed the expansion of the ERM-like proteins within the vertebrate clade, which occurred after its separation from Urochordata (*Ciona intestinalis*). Amino acid sequence alignment reveals the absence of an actin-binding site in the C-terminal region of all merlin proteins from various species but the presence of a conserved internal binding site in the N-terminal domain of the merlin and ERM proteins. In addition, a more conserved pattern of amino acid residues is found in the region containing the so-called "Blue Box," although some amino acid substitutions in this region exist in the merlin sequences of worms, fish, and *Ciona*. Examination of sequence variability at functionally significant sites, including the serine-518 residue, the phosphorylation of which modulates merlin's intra-molecular association and function as a tumor suppressor, identifies several potentially important sites that are conserved among all merlin proteins but divergent in the ERM proteins. Secondary structure prediction reveals the presence of a conserved α-helical domain in the central to C-terminal region of the merlin proteins of various species. The conserved residues and structures identified correspond to the important sites highlighted by the available crystal structures of the merlin and ERM proteins. Furthermore, analysis of the merlin gene structures from various organisms reveals the increase of gene length during evolution due to the expansion of introns; however, a reduction of intron number and length appears to occur in the merlin gene of the insect group.

**Conclusion:**

Our results demonstrate a monophyletic origin of the merlin proteins with their root in the early metazoa. The overall similarity among the primary and secondary structures of all merlin proteins and the conservation of several functionally important residues suggest a universal role for merlin in a wide range of metazoa.

## Background

The advancement in genome sequencing projects, the accumulation of knowledge in bioinformatics, and the molecular genetic analysis of genes and their functions in a variety of model organisms provides us with an unprecedented opportunity to identify novel genes based on sequences related to characterized genes [1]. This process is conducted using pairwise sequence comparison with the understanding that genes form families wherein related sequences likely share similar functions. Although initial identification of new genes may not yield a clear indication of their respective functions, studies on their evolution may allow validation of their sequence identity and provide information on their putative functional characteristics. For genes evolved from duplication and/or adapted to different evolutionary niches during speciation, detailed sequence comparison can provide additional information regarding their biological and biochemical characteristics [2].

Neurofibromatosis type 2 (NF2) is a highly penetrant, autosomal dominant disorder, whose hallmark is the development of bilateral vestibular schwannomas [3,4]. The tumor suppressor gene associated with NF2 has been identified and termed the *neurofibromatosis type 2 *gene (*NF2*) [5,6]. The *NF2 *gene encodes a protein named merlin, for moesin-ezrin-radixin like protein, or schwannomin, a word derived from schwannoma, the most prevalent tumor seen in NF2. For simplicity, we refer to the *NF2 *gene product as merlin hereafter.

Merlin shares sequence similarity with the ezrin, radixin, and moesin (ERM) proteins, which belong to the protein 4.1 superfamily of cytoskeleton-associated proteins that link cell surface glycoproteins to the actin cytoskeleton [7,8]. Like ERM proteins, merlin consists of three predicted structural domains [5,6,9]. The N-terminal domain, termed the FERM (F for 4.1) domain, is highly conserved among all members of the ERM family and is important for interactions with cell surface glycoproteins, including CD44 and intercellular adhesion molecules [10-13]. Crystal structure analysis shows that the tertiary structure of the FERM domain of merlin closely resembles those of the FERM domain of moesin and radixin [14-18]. The FERM domain of merlin exists as a clover-shaped molecule consisting of three structural subdomains A, B, and C, which are homologous to lobes F1, F2, and F3 in moesin and radixin. Subdomain A, composed of residues 20–100, possesses a ubiquitin-like fold. Subdomain B, consisting of residues 101–215, folds itself into a topology like that of the acetyl-CoA-binding protein. Subdomain C, containing residues 216–313, adopts the pleckstrin homology/phosphotyrosine-binding fold found in a broad range of signaling molecules [14-16]. The second half of merlin contains a predicted α-helix domain, which is also present in the ERM proteins [19]. Although the unique C-terminus of merlin lacks the conventional actin-binding domain found in the ERM proteins [20,21], merlin can directly bind actin using the residues at the N-terminal domain and indirectly through its association with βII-spectrin or fodrin [22-24].

The merlin and ERM proteins are thought to be key regulators of interactions between the actin cytoskeleton and the plasma membrane in polarized cells. They act as important members of signal transduction pathways that control cell growth and participate in the sorting of membrane proteins during exocytic traffic [25,26]. However, unlike the ERM proteins, merlin has a distinct function as a tumor suppressor [27]. Growth suppression by merlin is dependent on its ability to form intramolecular associations [28,29]. In this regard, merlin exists in an "open" (inactive form) or "closed" (active growth-suppressive form) conformation that is regulated by phosphorylation [30-35].

While previous studies have focused primarily on the functional analysis of merlin, limited information is available about its overall distribution across eukaryotes and its evolution. A phylogenetic study indicates that the FERM domains of ERM homologs from sea urchins, *Caenorhabditis elegans*, *Drosophila melanogaster*, and vertebrates share 74–82% amino acid identity and have about 60% identity with those of merlin [25,36-42]. These levels of identity are exceptionally high, suggesting that the protein structures of the merlin and ERM proteins from these species may be well-conserved. The most divergent ERM proteins are found in tapeworms and schistosomes [36-39]. The FERM domains of these parasite proteins share only 44–58% similarity to their vertebrate homologs. The high degree of structural conservation among these proteins points to possible similarities or functional redundancies. Intriguingly, no FERM domain-encoding genes have been identified in the genome of the yeast *Saccharomyces cerevisiae*, implying that FERM domains evolved in response to multicellularity, rather than as a cytoskeletal component [25].

The goal of the present study was to expand our understanding of the taxonomic diversity of merlin and the phylogenetic relationships using experimentally annotated and predicted sequences. By the integration of the BLAST-based analysis using the available partial and whole genome sequences with phylogeny reconstruction, we have generated an evolutionary tree for the entire ERM-family members from various taxa and identified some interesting details about their phylogenetic origin. In addition, we compared sequence variability at functionally significant sites, including the major phosphorylation site of merlin, predicted the secondary structure of the merlin proteins of various species, and examined the exon-intron structural evolution of the *NF2 *gene.

## Results and Discussion

### BLAST identification of merlin sequences

To identify putative merlin and ERM sequences in a wide range of eukaryotes, we performed BLAST analysis of 15 available genome databases. By searching through all annotated proteins and genome sequences, we identified 50 sequences from 30 species. Table 1 summarizes the full list of the predicted and annotated merlin and ERM proteins identified, and their GenBank and available UniProtKB/Swiss-Prot accession numbers and related resources. No merlin-like sequences were found in the genomes of fungi, plants, and protozoa. While the sequencing projects of the hard ticks are still ongoing at The Institute for Genomic Research (TIGR), amino acid sequences deduced from partial cDNAs of salivary glands, which share a similarity with the FERM domain of merlin, have been noted from *Rhipicephalus appendiculatus *[43], *Amblyomma variegatum *[44], and *Boophilus microplus *[45].

**Table 1:** The list of the predicted and experimentally annotated merlin and ERM proteins included in this study.

Species	Proteins	UniProtKB/Swiss-Prot Identifiers	GenBank Accession No.	Entries from Genome Sequencing Projects	Related Resources
***Homo sapiens***	merlin (NF2)	P35240	AAA36212		
	ezrin	P15311	CAA35893		
	radixin	P35241	AAA36541		
	moesin	P26038	AAA36322		
***Pan troglodytes***	similar to NF2		XP_515061		
***Papio anubis***	merlin	P59750	AAO23133		
***Bos taurus***	ezrin	P31976	AAA30510		
***Sus scrofa***	radixin	P26044	AAB02865		
	moesin	P26042	AAB02864		
***Canis familiaris***	similar to NF2		XP_534729		
***Oryctolagus cuniculus***	ezrin	Q8HZQ5	AAN06818		
***Mus musculus***	ezrin	P26040	CAA43086		
	radixin	P26043	CAA43087		
	merlin	P46662	CAA52737		
***Rattus norvegicus***	ezrin	P31977	AAR91694		
	NF2		XP_341249		
***Gallus gallus***	ezrin	Q9YGW6	BAA75497		
	radixin	Q9PU45	CAB59977		
	merlin		NP_989828		
***Xenopus laevis***	unknown		AAH77822		
	protein				
***Danio rerio***	nf2a	Q6Q413	AAS66973		
	moesin	Q503E6	AAH95359		
***Fugu rubripes***	radixin			FRUP00000132603	
	moesin			FRUP00000156313	
	merlin			FRUP00000136298	
***Tetraodon nigroviridis***	unnamed		CAG08868		
	protein 1		CAG08250		
	unnamed				
	protein 2				
***Ciona intestinalis***	erm-like			ci0100149701	
	merlin-like			ci0100130636	
***Ciona savignyi***	merlin-like			paired_scaffold_109	
***Biomphalaria glabrata***	erm-like		AAK61353		
***Lytechinus variegates***	moesin	P52962	AAC46514		
***Apis mellifera***	similar to schwannomin		XP_392673		
***Drosophila melanogaster***	merlin	Q24564	AAB08449		
	moesin	P46150	AAB48934		
***Drosophila yakuba***	merlin-like			predicted in this work	
***Anopheles gambiae***	merlin-like fragment		EAA07087		
***Caenorhabditis elegans***	erm1a	P91015	AAB37643		
	erm1b	P91016	AAB37642		
	nfm 1a	Q20307	AAA19073		
	nfm 1b	Q95QG5	AAK68385		
***Caenorhabditis briggsae***	erm-like			BP:CBP03133	
	nfm1			BP:CBP05025	
***Caenorhabditis remanie***	merlin-like			predicted in this work	
	erm-like				
***Brugia malayi***	merlin-like			316.m00022	
***Schistosoma japonicum***	JF2		AAB49033		
***Taenia saginata***	myosin-like	Q94815	CAA65728		
***Echinococcus multilocularis***	EM10		A45620		
***Echinococcus granulosus***	EG10	Q24796	CAA82625		
***Phanerochaete chrysosporium***	---				
*Aspergillus flavus*	---				
*Arabidopsis *thaliana	---				
***Oryza sativa***	---				
***Trypanosoma brucei***	---				
***Cryptosporidium parvum***	---				

### Assembly of predicted merlin sequences from whole genome shotgun

To date, the genomes of *Caenorhabditis remanei and Drosophila yakuba *are represented by a set of contigs [46]. When contigs are ordered, oriented, and positioned with respect to each other by mate-pair reads, they are described as a scaffold. Scaffolds are the main product of the Whole Genome Shotgun strategy and can be assigned to chromosomes using chromosome-specific markers. Although the extensive scaffolds for the genomes of *Caenorhabditis remanei *and *Drosophila yakuba *are not currently available, we were able to assemble predictive protein sequences, which most resemble the merlin sequence of the closely-related organism, *Caenorhabditis elegans *or *Drosophila melanogaster*, respectively, using TBLASTN search across the available sets of contigs. In the *Drosophila yakuba *contig 49.37, we identified a predicted merlin sequence, which is nearly identical to that of the *Drosophila melanogaster *protein with the exception of three positions at the C-terminus, two substitutions at Glu^468^→Asp and Asn^579^→Ser and an insertion of Lys at position 575. Also, we found three *Caenorhabditis remanei *contigs, 564.6, 2151.1, and 2151.2, which contained merlin-like sequences with similarity, ranging from 81% to 100%, to its *Caenorhabditis elegans *counterpart. It should be noted that the deduced amino acid sequences were assembled manually, and in some cases, only partial or approximate amino acid sequences could be obtained. Nevertheless, they were useful for the identification of the definite gene in the respective genome and were valuable for the following phylogenetic reconstruction in order to validate the functional relationship and evolution of the definite gene.

### Construction of a phylogenetic tree for the ERM family of proteins

**Figure 1:**
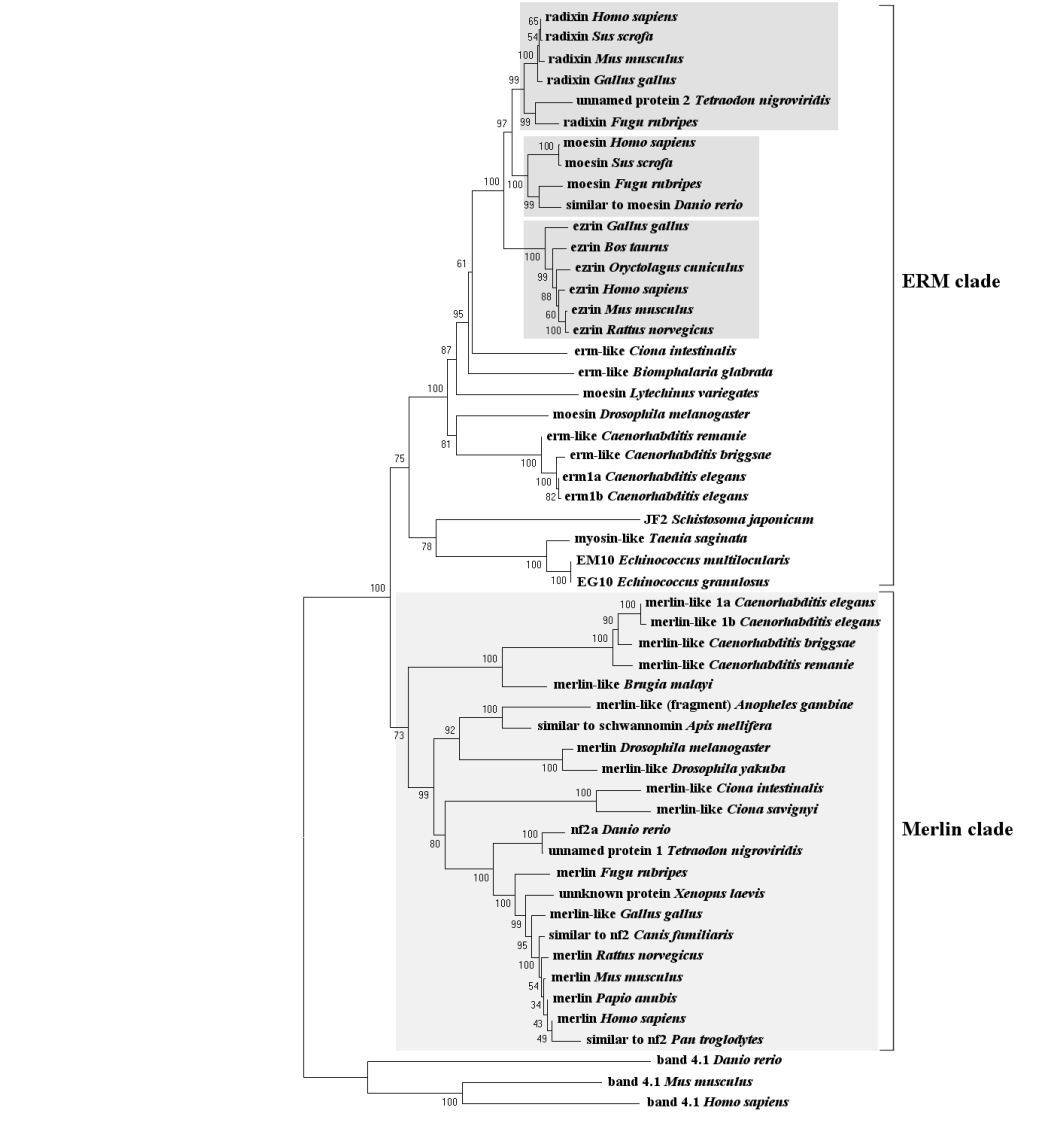
**The neighbor-joining tree of the ERM family**. The diagram illustrates the basic resolution of the ERM-family members into two major clades, merlin and ERM. Bootstrap support values are shown above each node. Shaded boxes denote different subgroups of the ERM clade in vertebrates, which appeared after the expansion of the ERM-like ancestor. The *Tetraodon nigroviridis *"unnamed protein 1 and 2" sequences (GenBank Accession No. CAG08868 and CAG08250, respectively) and the *Xenopus laevis *"unknown protein" sequence (GenBank Accession No. AAH77822) were grouped based on their similarity to the merlin or ERM sequences.

To understand the origin and evolution of merlin, we conducted a phylogenetic analysis of the 50 proteins of the ERM family, which were identified from 30 different taxa (Table 1) using the neighbor-joining method [47,48] combined with the molecular evolutionary genetics analysis program MEGA2 [49]. Three protein 4.1 sequences from humans, mice, and zebrafish, respectively, were used as an outgroup. By comparing the bootstrap support values, which denote the number of times a grouping occurs out of 1,000 random samples from the alignment, we constructed a phylogenetic tree for the ERM family of proteins (Figure 1). Based on this phylogenetic analysis, the entire ERM family can be subdivided into the ERM clade and the merlin clade. While both clades show a strongly supported monophyletic origin, the merlin clade can be robustly defined and separated from the ERM clade (the bootstrap support value = 100). We identified a total of 22 sequences for the merlin clade and 28 sequences for the ERM clade. The topology of the phylogenetic tree within the merlin clade appears to agree with the general concept of evolutionary history of speciation.

The merlin clade can be further divided into three groups according to the order of derivation: worms, insects, and Chordata, with the earliest separated genus, *Ciona*, in the last taxonomic unit (Figure 1). The predicted merlin-like sequence from *Caenorhabditis remanei *branched from that of *Caenorhabditis elegans*, and similarly, *Drosophila yakuba *diverged from its *Drosophila melanogaster *counterpart. Both the "unnamed protein 1" of *Tetraodon nigroviridis *and the "unknown protein" of *Xenopus laevis *from the GenBank database are clustered in the Chordata merlin-like group with high bootstrap probabilities (Figure 1), which confirms their identity as merlin homologs. The protein fragment from *Anopheles gambiae*, which bears a sequence similarity to merlin, is grouped together with the *Apis mellifera *merlin-like protein by a bootstrap support value of 100.

Although the ERM-like proteins have been identified in *Taenia saginata*, *Schistosoma japonicum*, *Echinococcus granulosus*, and *Echinococcus multilocularis *[36-39], we did not find any merlin-like sequences in the genomes of these species. The lack of merlin-like sequences in these parasite genomes may be due to incomplete genome sequences in the database; however, this explanation is unlikely because the merlin-like sequence was also not observed in the genome of *Schistosoma mansoni*, which has been rigorously studied [50]. Another possibility is that the absence of merlin-like sequences in these organisms may reflect their adaptation to a parasitic lifestyle and the reduction of various organ systems. Alternatively, the merlin protein may emerge later during evolution. Similarly, no merlin-like sequence was found in the complete genomes of protozoa, fungi, and plants. Based on these results, we suppose that the derivation of merlin occurred in the early metazoa after its separation from flatworms.

As illustrated in the ERM clade in Figure 1, the ERM-like proteins found in parasites can be grouped together but form a separate branch from the rest of ERM proteins. Based on the phylogenetic analysis, the clustering of the "unnamed protein 2" of *Tetraodon nigroviridis *with the *Fugu rubripes *radixin protein defines it as a radixin-like protein. It should be noted that the two predicted ERM proteins, erm1a and erm1b of *Caenorhabditis elegans *[51], may represent different transcript variants of the same gene (also see below).

Furthermore, we have observed the evident expansion of the ERM-like ancestor in vertebrates (Figure 1). Since the ERM homolog of *Ciona *emerged prior to the vertebrate clade, it appears that the first duplication of the vertebrate ERM sequence occurred after its divergence from *Ciona*. Subsequent expansion within this sub-family has led to the present existence of three related groups of proteins, ezrin, radixin and moesin; among which, the ezrin group is the most ancient. Such an expanded complement may only be common to the ERM proteins of vertebrates because other metazoa have only one predicted ERM-like homolog [52-56]. Curiously, the increasing number of ERM members that occurred within the vertebrate clade paralleled the evolutionary complexity of the organism. It will be important to understand how these proteins evolved and how their functions coordinated because of the important and diverse functions of ERM proteins [8,25,26].

### Evolution of the functionally important residues in merlin

**Figure 2:**
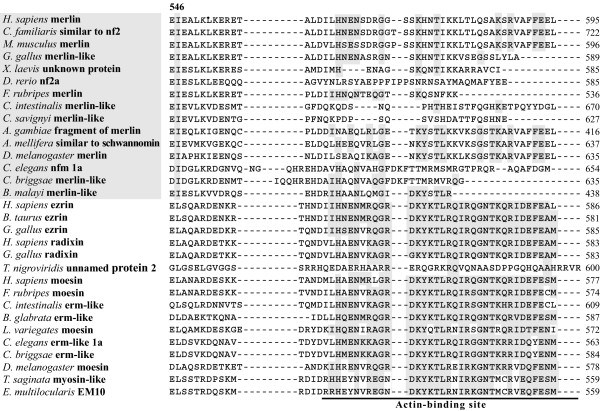
**Sequence alignments of functionally important sites in the merlin and ERM proteins of various species**. Comparison of the C-terminal region including the actin-binding site and two other predicted significant residues. Databank resources for the ERM-family proteins listed in Table 1 were used in the analysis, and only typical representatives from each group are displayed.

**Figure 3:**
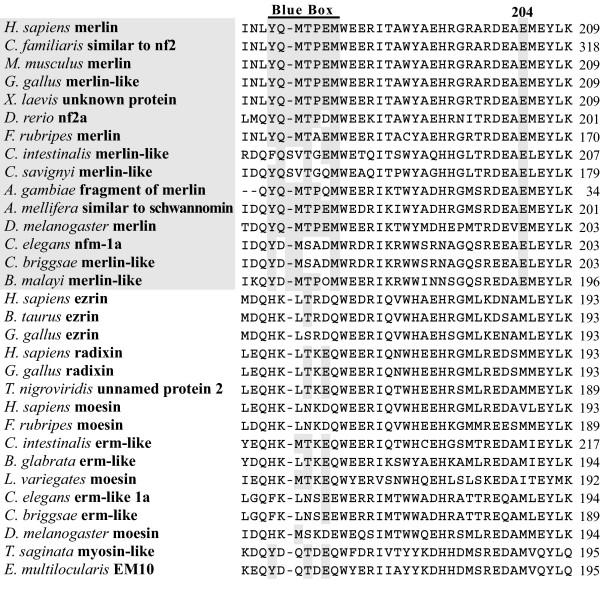
Alignment of the N-terminal domain, containing the Blue Box and the amino acid residue 204, conserved among the merlin proteins but divergent in the ERM proteins.

**Figure 4:**
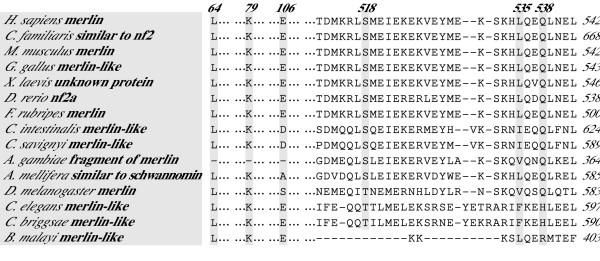
Sequence alignments reveal conservation of several functionally important residues, including the major phosphorylation site of the merlin group.

Although initial identification of proteins via sequence similarities does not yield a clear indication of their respective functions, analysis of specific conserved regions and residues may provide important information regarding their putative functional characteristics. We conducted pairwise sequence comparison among all obtained merlin and ERM sequences, and identified several regions of interest. The results of the entire sequence alignment are provided in the Additional File 1 and are summarized in Figures 2, 3, and 4. Previously, the conservation of the N-terminal FERM domain among human ERM proteins and their functional importance were described [10-13]. In our alignment, we showed that this conservation extended to the merlin and ERM proteins of various species for which sequences were available to date. These data suggest a universal role for the presence of the FERM domain during evolution and further imply an existence of certain evolutionary constraints on the changes of their amino acid residues.

Although merlin lacks the C-terminal actin-binding site found in ERM proteins [7,20,21,57], it can directly interact with the actin cytoskeleton [22,58] or indirectly bind via the actin-binding protein βII spectrin/fodrin [23,24]. Sequence alignment showed extensive amino acid variability in the C-terminal region of the merlin proteins of various species, while a noncontiguous stretch of 25 amino acid residues, including the well-defined actin-binding site, was reliably aligned among all predicted ERM proteins with the exception of the "unnamed protein 2" of *Tetraodon nigroviridis *(Figure 2). According to the phylogenetic tree, the "unnamed protein 2" of *Tetraodon nigroviridis *is classified in the radixin group (Figure 1), and its sequence visibly differs from other radixin proteins only at the C-terminus. The reason for this sequence variability is presently unknown. It may be due to an inaccuracy in sequence assembly from the scaffold. Alternatively, the "unnamed 2 protein" may possess a unique characteristic and will be of considerable interest for functional comparison with other radixin proteins.

Sequence variability at the C-terminal domain of the merlin proteins of various species appears to be high, while some conservation can be found within separate taxonomic groups such as vertebrates, insects, and worms (Figure 2 and Additional File 1). A part of the C-terminal region is absent in *Fugu rubripes*, *Danio rerio*, *C. briggsae*, and *Brugia malayi*. This may be due to partial assembly of the protein sequences, as all of them were predicted by bioinformatics using the available genomes and cDNA sequences. Alternatively, the lack of conservation in the C-terminal region of merlin in these species may imply that this region does not share the same function. In the remaining organisms, the C-terminal amino acid residues have a specific charge distribution, in spite of decreased hydrophilicity, when compared with the C-terminal part of moesin [15]; however, they likely form structures similar to the B, C, and D helices found in moesin.

Unlike ERM proteins, two regions (residues 1–27 and 280–323) in the N-terminal half of merlin have been mapped that are sufficient for binding to F-actin [59,60]. The first 17 amino acids in the N-terminus of human merlin are present in the merlin proteins of various species but not in any ERM proteins (see Additional File 1). The merlin proteins of higher vertebrates contain these residues, eight of which are absent in the merlin proteins of other organisms. Crystal structure analysis suggests that the structure of these extreme N-terminal residues of merlin is disordered in solution but likely becomes ordered as merlin binds to some effector targets [17]. Our sequence alignment indicates that the conservation in the extreme N-terminus of merlin extends to the first 27 residues. The distribution of specific positively-charged residues also appears to be conserved in this N-terminal portion of the merlin proteins of lower vertebrates and insects. These results suggest that the first 27 amino acids of merlin serve as a common protein-binding motif. It is noteworthy that a similar sequence can be found in the ERM-like protein of *Ciona*; however, the N-terminal region of the *Ciona *protein contains ten positively-charged, basic amino acids, which may affect the binding to actin and/or other proteins (Additional File 1).

The internal actin-binding site, containing residues 280–323 in the N-terminal half of merlin, was found to be highly conserved among all merlin and ERM proteins analyzed, particularly the last 30 amino acid residues (Additional File 1). This region contains an extended helix at the beginning of the α-helical domain and its importance is supported by the identification of several disease-causing mutations (S315F, L316F, L316W, Q324L), which were predicted to destabilize the α-helical segment and disrupt its hydrogen bonding with subdomain A [16-18]. In addition, these residues have been shown to associate with F-actin in moesin [61,62] and to contribute to the ICAM-2-binding site in radixin [14].

Previously, LaJeunesse et al. [63] identified seven functionally important amino acid residues (^170^YQMTPEM^177^) in the N-terminal domain of *Drosophila *merlin, called the "Blue Box." These seven amino acids are identical between the human and *Drosophila *merlin proteins but differ from the ERM proteins. Sequence comparison revealed a more conserved pattern of the Blue Box; all seven amino acid residues of the Blue Box were found to be identical in the merlin sequences from vertebrates, fruit flies, and honeybees (Figure 3); however, several amino acid substitutions were found in the Blue Box of worms, fish, and *Ciona*. The most interesting substitutions were found in the merlin-like protein of *Caenorhabditis *from ^174^ThrProGlu^176 ^to ^174^SerAlaAsp^176^. It is noteworthy that the methionine residue at position 177 in the Blue Box is conserved among all merlin proteins but not in the ERM proteins. These results further corroborate the functional importance of the seven amino acids in the Blue Box [63].

According to the crystal structure of the FERM domain in human merlin, the Blue Box residues are located in helix α3B of subdomain B [18] and form a defined area that is located on the surface of the protein [17]. Intriguingly, the three-dimensional conformation of merlin's Blue Box region is similar to that of the equivalent region in radixin [18], suggesting that regions in addition to the Blue Box are required for merlin to function as a tumor suppressor. Note that regions closely adjacent to the Blue Box-equivalent residues in human ERM proteins have been shown to participate in the N-terminal to C-terminal intramolecular interaction and ligand-binding, enabling increased mobility and structural changes in the activated FERM domain [14-16,64]. In light of the functional importance of the Blue Box in *Drosophila *merlin, its sequence conservation during evolution, and its location on the surface of merlin, the Blue Box probably participates in specific protein-protein interactions and contributes to other activities of merlin.

As in ERM proteins, phosphorylation affects the subcellular localization and intra- and inter-molecular associations of merlin [13,30-32]. In addition, it modulates the ability of merlin to suppress cell growth [34,35]. Two phosphorylation sites have been mapped to the Ser^518 ^and Thr^576 ^residues in the merlin protein. Phosphorylation on Ser^518 ^has been shown to modulate the ability of merlin to form intramolecular associations and to bind to critical effectors important for growth suppression [34,35]. In contrast, phosphorylation on the Thr^576 ^residue has no effect on merlin's functional activity, while phosphorylation on this residue is important for the function of ERM proteins [57,65-67]. Sequence alignment shows that the Ser^518 ^residue is conserved across all merlin proteins of various species with the exception of the fruit fly and the worm, which contain a related threonine residue at the corresponding position (Figure 4). Since both the serine and threonine residues can be phosphorylated, we suggest that the corresponding threonine residue in merlin proteins of the fly and the worm may act as a phosphorylation site.

Gutmann et al. showed that mutations within the predicted α-helical region of the human merlin protein had little effect on its function, whereas those in its N- or C-terminus rendered the protein inactive as a negative growth regulator [28,29]. Specifically, five naturally occurring missense mutations, L64P, K79E, E106G, L535P and Q538P, were found to inactivate merlin function. Interestingly, we found that the Leu^64 ^and Lys^79 ^residues were conserved among the merlin and ERM proteins of various species (Figure 4). According to the crystal structure of the FERM domain of merlin, the L64P substitution would create a cavity in the hydrophobic core of subdomain A and affect its β-sheet structure [17,18]. The significance of this structural information was further supported by the finding that the L64P mutation impaired the ability of merlin to form an intramolecular complex between its two N-terminal interaction sites [28]. Moreover, the L64P mutant lost its ability to bind the cytoplasmic tail of CD44; this interaction correlates with the ability of merlin to function as a growth suppressor [29].

The Lys^79 ^residue is situated at the end of helix α4A, and mutation at this residue (K79E) may cause the formation of a salt bridge with its neighboring Lys^76 ^residue, which is normally hydrogen bonded to Tyr^66 ^in helix α3A [17]. Two equivalent lysine residues, Lys^60 ^and Lys^63^, were found in module F1 of moesin and were predicted to be involved in specific protein interactions, consequently changing the structure of an activated molecule [15,16]. Together with a group of positively-charged amino acids at the beginning of the helix module F3 (R275, K278, R279), these lysine residues interact with the negatively-charged residues (342-REKEE-346) in the C-terminal region [16]. Importantly, most of the homologous positively-charged residues located between lobes F1 and F3 in the radixin protein have been shown to bind to inositol 1,4,5-trisphosphate (IP3) [15]. In addition to Lys^79^, the Lys^76 ^residue was also found to be highly conserved among various merlin and ERM proteins with the exception of the worm protein, which has a Gln^76 ^instead of Lys^76 ^(Figure 4 and Additional File 1). Also, the ERM-like proteins of parasites *Taenia saginata*, *Echinococcus granulosus*, and *Echinococcus multilocularis *contain an Arg^76 ^residue, which is also a basic amino acid residue and may be capable of participating in interactions similar to those of the corresponding lysine residue. On the contrary, in the JF2 protein of *Shistosoma mansoni*, the position equivalent to Lys^60 ^of moesin is occupied by a glutamic acid residue, and no conservation of residues 275, 278, and 279 in the JF2 protein was found, suggesting a unique structural feature for this S*histosoma *protein.

Several other naturally-occurring missense mutations on human merlin, including E106G, L535P and Q538P, have also been found to affect its functional activity [29,68]. Our sequence alignment revealed that the Glu^106^, Leu^535^, and Gln^538 ^residues were conserved among the merlin proteins of the Chordata group (Figure 4), highlighting the general importance of these residues for merlin function. Similar to the L64P mutation described above, the E106G mutation resulted in impaired intramolecular associations of merlin [29]. However, the Leu^64 ^residue is highly conserved among all merlin and ERM proteins of various species, while Glu^106 ^is conserved only in the merlin proteins of Chordata and worms. In the crystal structure of the FERM domain of merlin, the Glu^106 ^residue is located at the linker A-B (α1'B) and participates in the inter-subdomain interaction by forming a hydrogen bond with the Lys^289 ^residue [17,18]. This interaction may enable subdomain B to rotate closer to subdomain C. Intriguingly, Lys^289 ^is conserved only among the merlin proteins of mammals, chickens, frogs, honeybees, and mosquitoes (Additional File 1). In the merlin proteins of other species, a negatively-charged aspartic or glutamic acid occupies this position, except in fish. Instead of lysine, an arginine residue was found in the homologous position of all ERM proteins (e.g., Arg^273 ^for moesin), except for the ERM-like proteins in parasites (Additional File 1). This Arg^273 ^residue, located at the beginning of the helix of subdomain F3, lies in a specific cleft between subdomains F1 and F3 with the positively-charged R275, K278, and R279 residues. According to the structure of radixin, the IP_3_-binding site is located at this basic cleft [14]. This region in the moesin protein has also been shown to interact with its C-terminal domain [16].

It should be mentioned that residues that are conserved in the merlin proteins, but not in the ERM proteins, of various species may be important for elucidating the functional difference between the merlin and ERM proteins. We found that the glutamic acid residue at position 204 of human merlin was conserved among all merlin proteins (Figure 3). In contrast, variable and uncharged amino acids were found at the corresponding position of the ERM proteins. Crystal structure of the FERM domain of human merlin shows that the Glu^204 ^residue lies in the beginning of helix α4B and is included in a specific stretch of amino acids in subdomain B [17]. By sequence alignment of human merlin and ERM proteins, about 70 amino acids, including this specific stretch of residues, which are unique to merlin but different in ERM proteins, were identified (see Additional File 1). The majority of these amino acids can be subdivided into three clusters; each of them is specific to its corresponding subdomain and is located on the surface of the protein. These results suggest that these 70 amino acids likely take part in protein-protein interactions. Note that the conserved stretch of amino acids in subdomain B also includes the functionally important "Blue Box" discussed above.

Similarly, the isoleucine residue at position 546 was found to be conserved among the merlin proteins of various species, while a leucine residue was present in the corresponding position in all ERM proteins (Figure 2). The residue homologous to Leu^529 ^in the C-terminal domain of moesin is located at the end of helix A with other hydrophobic residues, tightly contacting the hydrophobic faces of helices B and D of subdomain F2 [15]. Although the information about such an interaction in merlin is presently unavailable, additional crystal structure analysis should allow us to better understand the importance of this amino acid residue. In addition, it will be interesting to test whether mutations in the conserved amino acid residues identified in this study could affect protein function.

### Predicted secondary structure of merlin and comparative analysis of the predicted α-helical region

**Figure 5:**
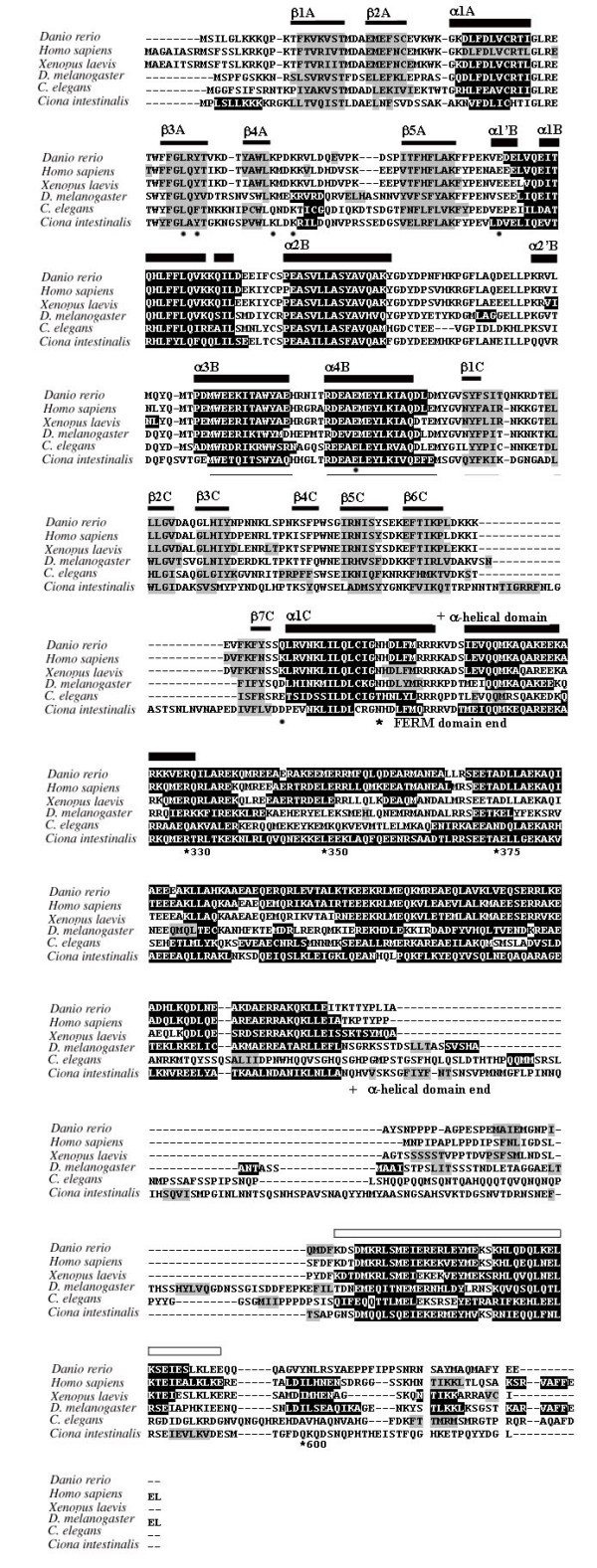
**The predicted secondary structures for the merlin proteins of various species**. The region with a predicted β-sheet structure is shaded in grey, while the region with an α-helix structure is shaded in black. These predicted secondary structures correspond to the crystal structural data [18], which are shown above the alignment with the α-helix region indicated with a thick black bar and the β-sheet region with a thin black bar. The predicted α-helical domain in the central-to-C-terminal region of merlin is marked with an open bar. Asterisks denote known domains of the merlin protein with numbers pointing to the end of truncated *Drosophila *merlin protein discussed in the text. "+" denotes the beginning and the end of the predicted α-helical domain. The positions of specific residues in the FERM domain discussed in the text are denoted by black dots below the aligned sequences.

Turunen et al. previously reported that the central region of ERM proteins contained approximately 200 residues that were predicted to be mostly α-helical [19]. To examine whether there was a similar structure present in all merlin proteins, we analyzed 21 merlin sequences from various organisms and predicted their secondary structures using the JPRED program [69]. The results of such an analysis for six representative species are presented in Figure 5. The predicted locations of α-helices and β-sheets in the N-terminal domain support the experimental findings from the structural analysis of the FERM domain of human merlin protein [18]. In addition, a predicted α-helical structure in the central-to-C-terminal region was found to be conserved among the merlin proteins of various species analyzed. Previously, it was shown that a truncated merlin protein of *Drosophila*, containing residues 1–600, lost the ability to localize to the cytoplasm and was concentrated at the plasma membrane [63]. However, two smaller truncated proteins, consisting of residues 1–350 or 1–375, were only loosely associated with the plasma membrane. These results suggest that the predicted α-helical region of merlin is important for its intracellular localization. Since almost the entire α-helical domain was absent in these truncated proteins, we suggest that it may contain a determinant for membrane association. This notion is further supported by the observation that additional truncated proteins, containing residues 1–300 or missing almost the entire α-helical domain, were diffusely localized to the cytoplasm.

In human merlin, the predicted α-helical structure is situated between residues K312 and K478 (Figure 5 and Additional File 1). The N-terminal border of this structure was clearly recognized for 21 merlin sequences analyzed, whereas the C-terminal boundary could be traced only up to Urochordata (*Ciona*) and Nematoda (*Caenorhabditis*). This α-helical domain, predicted from all 21 merlin sequences, contains a high density of charged amino acids (from about 25% in *Ciona *to greater than 40% in vertebrates). Sequence alignment reveals 19 conserved amino acid residues in this predicted α-helical domain (Additional File 1). The amino acid identity for the predicted α-helical domain within each phylogenetic group is as follows: 1) Genus *Drosophila *(*D. melanogaster *and *D. yakuba*) – 99% (one amino acid substitution), 2) Genus *Caenorhabditis *(*C. elegans*, *C. briggsae*, and *C. remanie*) – 85.7%, 3) Genus *Ciona *(*C. intestinalis *and *C. savignyi*) – 71.7%, 4) vertebrates – 63.5%, and 5) mammals – 90%. Taken together, these results indicate that the merlin proteins of various species contain a conserved α-helical domain in the central to C-terminal region.

### Exon-intron structural evolution of the merlin gene

**Figure 6:**
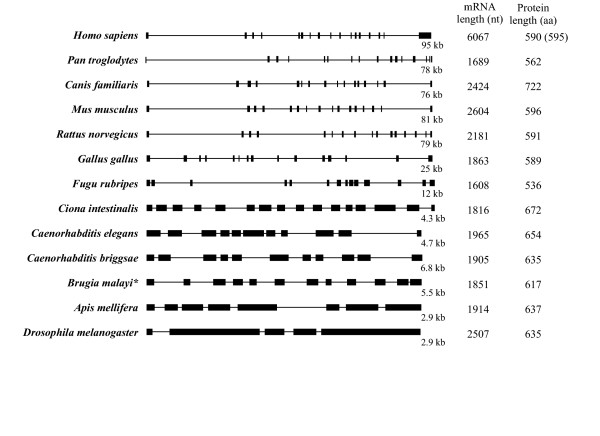
**Schematic diagram of the exon and intron structures for the merlin genes of various species**. The horizontal line depicts the merlin gene with its size indicated in base pairs (bp) on the right. The upright boxes represent exons. The lengths of the merlin mRNA sequences available in the database are shown in nucleotides (nt) and the lengths of the predicted merlin proteins are also indicated in amino acids (aa). The indicated human *NF2 *mRNA refers to the longest, full-length transcript identified, which contains a long 3' untranslated region [72]. Two major *NF2 *isoforms I and II are produced via alternative splicing and the lengths of their protein products are shown with that of isoform I indicated in the parentheses. It should be noted that Northern blot analysis detected the rat and mouse *NF2 *mRNAs of about 4.5 kb, indicating that the sizes of the rodent *NF2 *mRNAs shown here are not full-length. The asterisk (*) denotes the exon-intron structure of *Brugia malayi *predicted from this study.

Recent progress in automated computational analysis of partially and completely sequenced genomes using a gene prediction method and the analysis of expressed sequence tag (EST) has provided considerable opportunities not only to describe novel genes but also their exon-intron structures. Such an approach also allows the examination of the presence of different splicing variants/isoforms. To understand the evolution of the exon-intron structure of the merlin gene, we assembled all available *NF2 *gene-related sequences from various taxa. Using the sequences of proteins, mRNAs, and combined contigs [70], we established the structure of the merlin-like gene for *Brugia malayi*, which consists of 12 exons and 11 introns (Figure 6). Analogously, the homolog of the *NF2 *gene in *Caenorhabditis elegans *was found to contain 11 exons and 10 introns. It should be mentioned that the two *NF2*-like sequences, nfm-1a and nfm-1b of *Caenorhabditis elegans*, differ from each other only by the sequence of the last exon (Additional File 1), suggesting that they represent cDNA isoforms.

**Figure 7:**
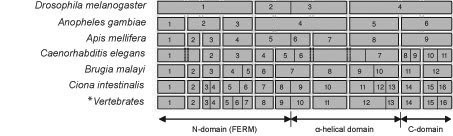
**The alignment of exons with specific domains of merlin reveals the presence of homologous introns**. Boxes represent the coding exons with numbers indicated accordingly. The locations of the three commonly discussed domains are marked with horizontal arrows under exons. The boundaries among these domains were defined according to the human merlin protein. The asterisk indicates that the exon structure shown is common to the merlin genes of all vertebrate species studied, including *Homo sapiens*, *Pan troglodytes*, *Canis familaris*, *Mus musculus*, *Rattus norvegicus*, *Danio rerio*, *Fugu rubripes*, *and Xenopus laevis*. The overall merlin gene structure of *Gallus gallus *is similar, except that exons 15 and 16 are fused together in this species.

As shown in Figure 6, the general arrangement of the merlin gene structure is conserved among mammalian species, especially at the region that encodes the N-terminal domain, albeit the length of the genes may differ slightly. The human *NF2 *gene consists of 17 exons and spans about 95 kb of DNA [5,6,71,72]. *NF2 *transcripts undergo alternative splicing, generating multiple isoforms [72-79]. Isoform I, missing exon 16, and isoform II, containing all 17 exons, are the two predominant species. As the result of alternative splicing, isoform 1 encodes a 595 amino acid protein. Isoform II differs from isoform I only at the C-terminus. Insertion of exon 16 into the mRNA provides a new stop codon, resulting in a 590 amino acid protein that is identical to isoform I over the first 579 residues. Because of the presence of a long 3' untranslated region, the longest *NF2 *isoform I RNA, containing the sequence from all 17 exons, is about 6.1 kb [72]. The merlin genes of other mammalian species, R*attus norvegicus*, *Canis familiaris*, *Mus musculus *and *Pan troglodytes*, contain 16 exons (Figures 6 and 7). In addition, alternatively spliced merlin isoforms have been found in rodent species [80-82]. On the contrary, the structure of the merlin genes of *Gallus gallus *and *Fugu rubripes *are arranged differently from those of mammalian species, with 15 and 14 exons spanning much shorter DNAs of only about 25 kb and 12.3 kb, respectively (Figure 6). Although the *NF2 *gene of *Fugu rubripes *has not yet been completely assembled, we predict that it lacks the sequences of the first and the last exons of the mammalian *NF2 *gene based on our sequence alignment (see Additional File 1).

In spite of the presence of 16 exons and the size of transcript similar to those found in some vertebrates, the gene for the merlin-like protein of *Ciona intestinalis *is relatively small with only about 4.3 kb (Figure 6). This tendency towards reduction of intron length and number continues to be seen in the merlin gene of worms and insects (Figures 6 and 7). The gene for the merlin-like protein of *Caenorhabditis elegans*, consisting of 11 exons, spans about 4.7-kb DNA, and that of *Brugia malayi*, containing 12 exons, is about 5.5 kb in length. The *NF2 *homolog of *Drosophila melanogaster *and the gene for the merlin-like protein of *Apis mellifera *are only about 2.9 kb, the smallest in the merlin clade, and consist of 5 and 8 exons, respectively (Figure 6). Interestingly, some conservation of the positions of homologous introns can be found in the *NF2 *gene from various species (Figure 7); however, the sizes of their introns are variable. Such an evolutionary conservation of homologous introns implies that the presence of regulatory sequences in the introns to regulate the transcriptional event.

Unlike the sizes and structures of the merlin or merlin-like genes in various organisms, the lengths of the merlin proteins and transcripts have not changed very much during evolution (Figure 6). Moreover, several functionally important domains of merlin also remain conserved. Since the merlin protein of the insect emerged after deviation from that of the worm, which was anciently derived from the common ancestor (Figure 1), it appears that the decrease in gene size and exon number occurred specifically within the insect group. This branch of merlin evolution is likely to develop independently and in the opposite direction from those more recently developed merlin proteins of Chordata. Parallel evolution towards the increase in merlin gene size and exon number between the worm and Chordata appears to be less likely. Thus, it is possible that the common ancestor for the merlin genes of the worm, the insect, and Chordata may contain a few more exons. During evolution, reduction of introns and/or fusion of exons occur within the insect group.

It is evident that the genome of the insect is more complicated than that of the worm. The simplification of the merlin gene structure in the insect is unique and may have a functional significance. This observation may explain the lack of splicing variants of the *NF2 *homolog in *Drosophila*, in contrast to those merlin isoforms found in mammals [72-82] and in *Caenorhabditis elegans *as we predicted in this study.

## Conclusion

We have conducted the phylogenetic analysis of merlin diversity across metazoan genomes using the experimentally annotated and predicted sequences in conjunction with bioinformatic tools. We show that the merlin proteins have a monophyletic origin with a root in the early metazoa. We have also established the expansion of the ERM-like ancestors within the vertebrate clade that occurred after its separation from Urochordata. Several potentially important sites that are conserved among all merlin proteins but are divergent in the ERM members have been identified. As supported by the crystal structure data, these conserved residues are likely to be important for merlin function. Analysis of the evolution of the merlin gene structure reveals the existence of common splicing variants in human and *Caenorhabditis elegans*. While a trend toward the increase of gene length during evolution was observed, a reduction of intron number and length appears to occur in the merlin gene of the insect group. Taken together, our results have important implications for the evolution of the merlin proteins and their possible functional variability in different taxa.

## Methods

### BLAST search

Initial sequences of genes and proteins of interest from various organisms were identified from the National Center for Biotechnology Information (NCBI) database [83] using the BLAST algorithm [84]. We then searched the desirable sequences across genomic databases of completely or partially sequenced genomes available at The Sanger Institute [85] and The Institute for Genomic Research (TIGR; [86]). Also, we investigated other available sequence databases that contain information for specific organisms. The sources of sequences of the predicted or experimentally annotated merlin and ERM proteins are summarized in Table 1.

To obtain the entire amino acid sequence of an annotated protein, we used UniProt from Universal Protein Resource [87]. The erythrocyte membrane proteins 4.1 sequences of *Homo sapiens *(GenBank: CAI21970), *Mus musculus *(GenBank: NP_001006665), and *Danio rerio *(GenBank: AAQ97985) were also included in the analysis as an outgroup. Because of the presence of many non-conserved and large introns in the genes of interest, we conducted BLAST search using TBLASTN alignment algorithm in the cases where no protein sequences were available.

### Alignments and phylogeny

The CLUSTAL_X program [88] was used to align the characterized or predicted protein sequences from different species. Phylogenetic analysis was carried out using the MEGA2.1 program [49].

### Secondary structure prediction

The secondary structure prediction program JPRED [69] was used to predict the secondary structure of each merlin protein from various species. This program uses physical-chemical properties of the amino acid sequence and neural network for recognition of α-helices and β-sheets.

## List of Abbreviations

NF2 – Neurofibromatosis type 2

*NF2 *– the *Neurofibromatosis type 2 *gene

ERM – ezrin, radixin, and moesin

FERM – 4.1, ezrin, radixin, and moesin

IP3 – inositol 1,4,5-trisphosphate

TIGR – The Institute for Genomic Research

EST, expressed sequence tag

NCBI – National Center for Biotechnology Information

## Authors' contributions

KG and AB carried out the phylogenetic analysis of merlin diversity across metazoan genomes and drafted the manuscript. EMA and LVO helped with the design of the study and preparation of data for the figures. LSC was the principal investigator of the project and participated in the design, coordination, and writing of the manuscript. All authors read and approved the final manuscript

## Supplementary Material

Additional File 1 **Complete amino acid sequence alignment of the merlin and ERM proteins**. Letters shaded in grey illustrate the conservation pattern of aligned sequences. The names of the merlin proteins from various species are shown in yellow. The conserved residues of the 'Blue Box' are also shaded in yellow. The positions of residues discussed in the text are colored in red. Blue letters denote the conserved residues within the predicted α-helical domain. Numbers indicated at the end of each sequence refer to the positions of the last residue within each protein analyzed. Click here for file
