# Supplementary material for: Evolution and origin of merlin, the product of the Neurofibromatosis type 2 (NF2) tumor-suppressor gene
Source: BMC Evol Biol. 2005 Dec 2;5:69. doi: 10.1186/1471-2148-5-69 (PMC1315344; doi:10.1186/1471-2148-5-69)
Supplement: Additional File 1 — Complete amino acid sequence alignment of the merlin and ERM proteins. Letters shaded in grey illustrate the conservation pattern of aligned sequences. The names of the merlin proteins from various species are shown in yellow. The conserved residues of the 'Blue Box' are also shaded in yellow. The positions of residues discussed in the text are colored in red. Blue letters denote the conserved residues within the predicted α-helical domain. Numbers indicated at the end of each sequence refer to the positions of the last residue within each protein analyzed. [file 1471-2148-5-69-S1.pdf]

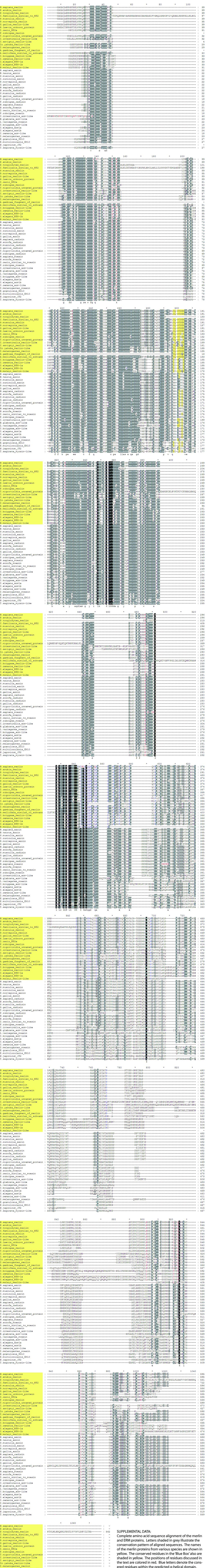

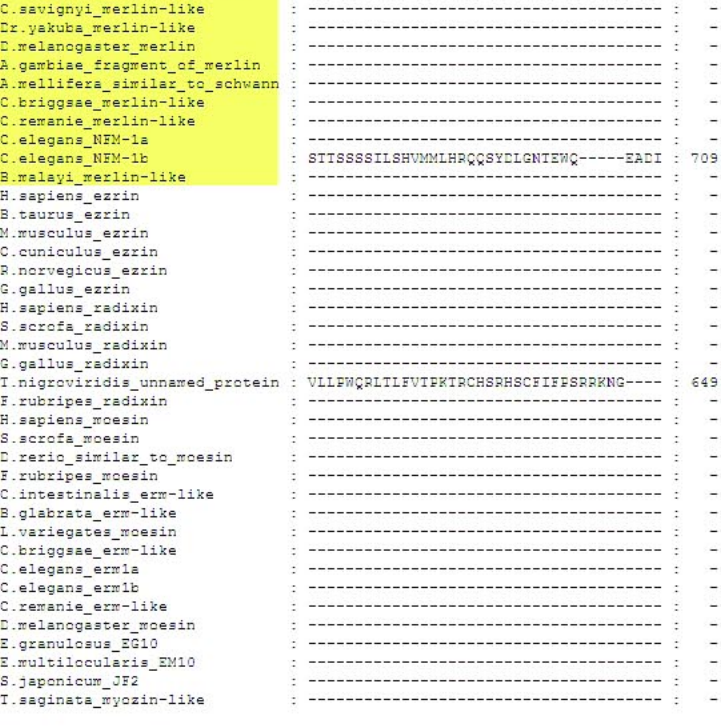

SUPPLEMENTAL DATA:
Complete amino acid sequence alignment of the merlin and ERM proteins. Letters shaded in grey illustrate the conservation pattern of aligned sequences. The names of the merlin proteins from various species are shown in yellow. The conserved residues in the 'Blue Box' also are shaded in yellow. The positions of residues discussed in the text are colored in red. Blue letters denote the conserved residues within the predicted α-helical domain. Numbers indicated at the end of each sequence refer to the positions of the last residue within each protein analyzed.
